# Trends in Electronic Health Record Inbox Messaging During the COVID-19 Pandemic in an Ambulatory Practice Network in New England

**DOI:** 10.1001/jamanetworkopen.2021.31490

**Published:** 2021-10-12

**Authors:** Bidisha Nath, Brian Williams, Molly M. Jeffery, Ryan O’Connell, Richard Goldstein, Christine A. Sinsky, Edward R. Melnick

**Affiliations:** 1 Department of Emergency Medicine, Yale University School of Medicine, New Haven, Connecticut; 2 Northeast Medical Group, Yale New Haven Health, Stratford, Connecticut; 3 Department of Emergency Medicine and Department of Health Care Policy Research, Mayo Clinic, Rochester, Minnesota; 4 American Medical Association, Chicago, Illinois; 5 Department of Biostatistics (Health Informatics), Yale School of Public Health, New Haven, Connecticut

## Abstract

This cross-sectional study compares patient electronic health record (EHR) inbox message volume in an ambulatory practice network in New England during the COVID-19 pandemic with prepandemic levels.

## Introduction

COVID-19–related disruptions have changed how patients access routine care. Anecdotal evidence indicates that, with the pandemic, ambulatory physicians experienced an increase in patient medical advice requests (PMARs). Therefore, we compared patient message volume during the pandemic with prepandemic levels.

## Methods

This cross-sectional study analyzed deidentified electronic health record metadata (Signal, Epic Systems) from March 2018 to June 2021 in a large ambulatory practice network in New England. This study was deemed exempt for review and informed consent by Yale University’s institutional review board because it involves secondary research of deidentified electronic health record metadata that did not include any patient identifier or private health information. This study follows the Strengthening the Reporting of Observational Studies in Epidemiology (STROBE) reporting guideline.

Trends in inbox message volume (categorized by message type and source; see eTable 1 in the Supplement), time in the inbox, visit volume and type (in-person vs telehealth), patient volume, and patient use of the patient portal were examined using descriptive statistics. Physician specialties were grouped into primary care, medical, and surgical specialties (eTable 2 in the Supplement). Variables were compared before (March 2018 to February 2020) and during (March 2020 to June 2021) the COVID-19 pandemic. To assess whether the onset of the pandemic was an inflection point in PMARs, message volume per physician per day was modeled by piecewise linear regression using a spline for month with a single knot at March 2020 and Huber-White SEs. Three months of inbox message data were missing (3 of 40 months [7.5%]) and excluded from the analysis. To test for significance (*P* < .05), we used a 2-sided Wald test for equivalence of the coefficients. We used Stata statistical software version 16 (StataCorp) for data analyses.

## Results

Forty months of inbox messages were analyzed, including 10 850 401 messages to 419 unique physicians from 38 specialties across 141 practice sites (Figure). Overall, primary care, medical, and surgical physicians received 49.3, 33.4, and 20.7 messages per day, respectively. Between March 2020 and June 2021, mean (SD) total messages per day increased from 45.0 (27.4) to 46.0 (27.4) messages per day for primary care physicians, from 29.3 (20.7) to 32.0 (20.8) messages per day for medical physicians, and from 16.6 (11.9) to 23.3 (17.9) messages per day for surgical physicians. Patient-originated messages also increased, including PMARs (from a mean [SD] of 1.8 [1.8] to 3.9 [3.2] messages per day for primary care physicians; from 1.0 [1.7] to 2.2 [2.9] messages per day for medical physicians; and from 0.4 [0.5] to 1.1 [1.3] messages per day for surgical physicians), patient calls, and time in the inbox (from 21.7 [12.7] to 25.1 [13.7] minutes per day for primary care physicians; from 13.4 [10.7] to 15.6 [9.2] minutes per day for medical physicians; and from 7.6 [7.2] to 11.1 [10.0] minutes per day for surgical physicians) (Table).

**Figure.**
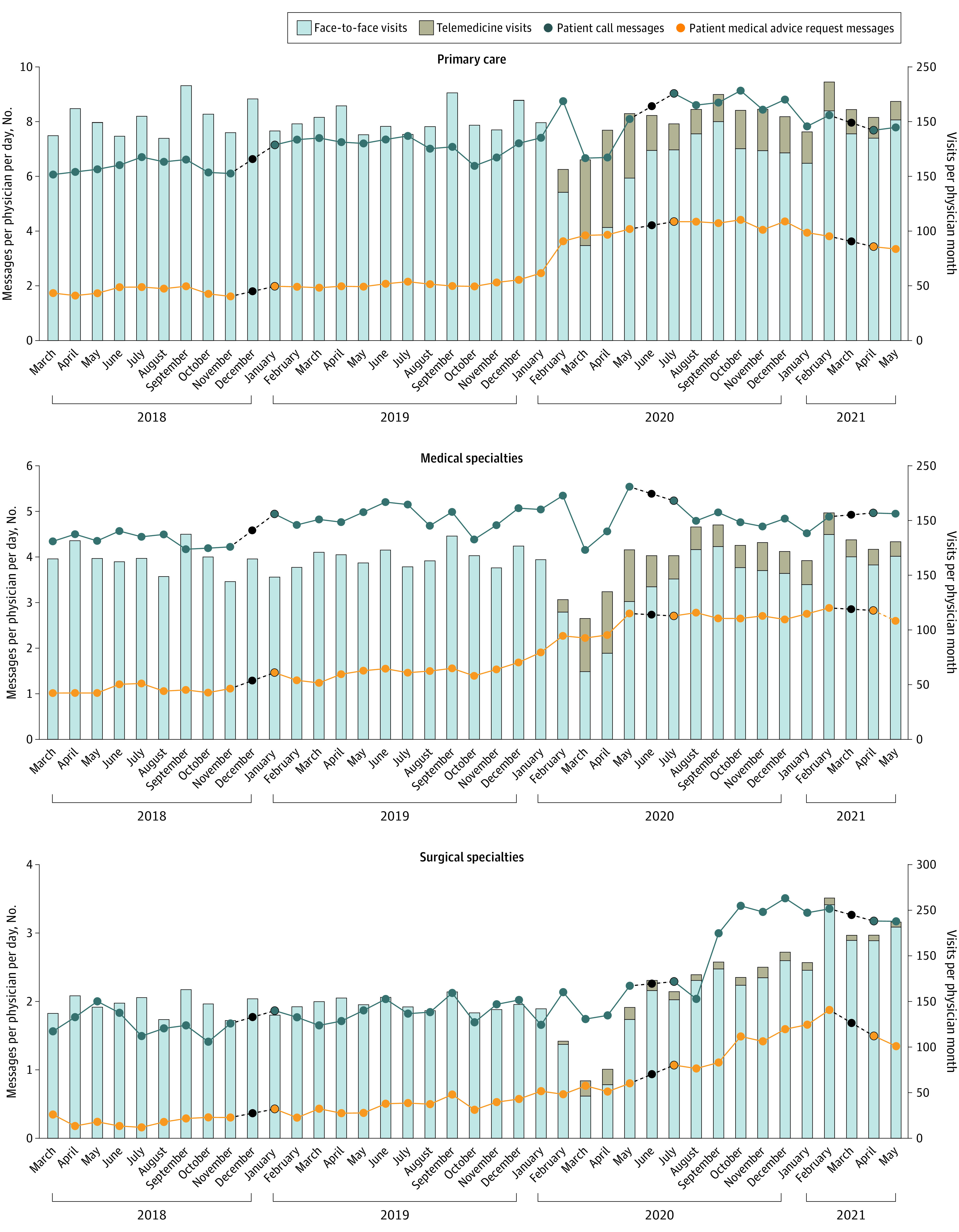
Raw Trends in Patient Medical Advice Requests and Patient Call Messages (Weighted Average, per Physician, per Day) by Specialty, Ambulatory Visit Volume, and Type, March 2018 to June 2021 Missing data are indicated by dashed lines and black dots. Findings are from modeling by piecewise linear regression of patient medical advice request trend using a spline for month with a single knot at March 2020 and Huber-White SEs.

**Table.** Inbox Message Metrics Before and During the COVID-19 Pandemic^a^

Variable	Primary care			Medical specialties			Surgical specialties		
	Before COVID	During COVID	Change, %	Before COVID	During COVID	Change, %	Before COVID	During COVID	Change, %
Message volume by category and inbox time per physician, per day, monthly weighted mean (IQR), No. of messages^b^									
Total messages^c^	45 (22-63.7)	46.0 (25.1-63.0)	2.2	29.3 (15.5-36.7)	32.0 (15.4-41.7)	9.2	16.6 (8.4-21.0)	23.3 (12.2-27.6)	39.9
Patient-originated messages (all categories)^c^	8.1 (3.3-11.2)	11.4 (6.7-16.7)	40.5	5.2 (1.5-8.1)	6.6 (2.0-9.8)	25.4	1.8 (0.2-2.5)	3.8 (0.6-4.7)	114.8
Patient medical advice request messages^c^	1.8 (0.5-2.4)	3.9 (1.2-5.1)	104.9^d^	1.0 (0.1-1.3)	2.2 (0.3-2.8)	107.7^d^	0.4 (0.1-0.4)	1.1 (0.3-1.3)	206.2^d^
Patient call^c^	6.5 (2.7-8.6)	7.8 (4.7-10.0)	21.1	4.2 (1.3-6.7)	4.4 (1.4-7.1)	4.0	2.2 (0.7-3.6)	2.7 (0.9-4.4)	19.4
Prescription renewal messages^c^	8.8 (1.9-13.3)	8.9 (2.0-13.4)	1.3	4.6 (1.2-6.9)	4.9 (0.9-8.2)	7.1	0.6 (0.1-0.5)	1.2 (0.1-1.3)	94.5
Physician user–initiated messages^c^	9.7 (4.8-13.9)	8.8 (4.4-11.0)	−9.0	6.4 (2.8-8.2)	5.9 (2.5-8.0)	−7.7	4.7 (2.1-6.2)	5.9 (2.3-8.1)	27.2
System-generated messages^c^	9.3 (4.7-11.8)	8.2 (4.4-10.9)	−11.7	7.2 (3.3-9.1)	8.0 (4.2-10.5)	11.1	7.2 (3.2-8.9)	4.5 (4.2-11.6)	−37.9
Team member–generated messages^c^	9.5 (5.1-12.4)	9.1 (5.1-12.0)	−4.3	6.4 (2.4-9.2)	6.9 (2.4-9.7)	7.9	2.6 (0.9-3.2)	4.5 (1.1-5.0)	74.7
Time in inbox, min^e^	21.7 (11.5-30.4)	25.1 (15.2-33.9)	15.6	13.4 (5.8-18.6)	15.6 (7.9-21.0)	16.6	7.6 (3.1-10.1)	11.1 (3.3-14.7)	46.1
Visit volume and type per physician, per month, mean (IQR), No.									
Total visits (encounters)	189.9 (128.8-257.3)	193.9 (130.7-253.6)	2.1	163.6 (95.9-220.4)	173.3 (109.3-234.5)	6.0	147.7 (95.5-175.1)	166.2 (93.1-211.7)	12.5
In-person visits	189.9 (128.8-257.3)	160.4 (109.0-218.8)	−15.6	163.6 (95.9-220.4)	147.2 (94.8-196.4)	−10.0	147.7 (95.5-175.1)	157.9 (93.1-197.4)	6.9
Telehealth visits	0 (0-0)	33.6 (16.2-46.0)		0 (0-0)	26.1 (5.5-36.2)		0 (0-0)	8.3 (0.4-8.6)	
Count of unique patients in visits and percentage with electronic patient portal account, weighted mean (IQR), per physician, per month									
Unique patients^f^	181.6 (123.9-243.6)	152.7 (109.5-207.8)	−15.9	157.2 (93.3-216.5)	143.3 (94.3-193.1)	−8.8	132.6 (82.8-156.7)	140.7 (82.3-186.1)	6.0
Patient MyChart utilization^g^	72.6 (68.1-81.6)	83.2 (79.1-90.2)	14.7	70.1 (63.0-77.3)	78.0 (73.5-86.0)	11.3	66.7 (56.6-76.4)	77.6 (71.7-84.9)	16.4
Unique physician count (N = 419)	223 (53.2)			142 (33.9)			54 (12.9)		
Message volume by category, count of messages, No. (%)^c^									
Total messages (all specialty combined = 10 850 401)	7 149 410 (65.9)			3 016 875 (27.8)			684 116 (6.3)		
Patient originated	1 489 406 (20.8)			579 996 (19.2)			85 972 (12.6)		
Prescription renewal	1 384 178 (19.4)			461 421 (15.3)			25 976 (3.8)		
Physician user initiated	1 437 779 (20.1)			606 487 (20.1)			182 306 (26.6)		
System generated	1 357 236 (19)			712 631 (23.6)			269 754 (39.4)		
Team member generated	1 480 811 (20.7)			656 340 (21.8)			120 108 (17.6)		

^a^ The period before the COVID-19 pandemic refers to March 3, 2018, to February 2020. The period during the pandemic refers to March 2020 to June 2021.

^b^ Refers to days in each reporting period that a clinician sees patients or logged into the electronic health record system.

^c^ See eTable 1 in the Supplement for message category details.

^d^ Findings from piecewise linear regression of patient medical advice request trend are shown in the Results section of the text.

^e^ Time in in basket per day (Signal, Epic Definition) refers to the mean amount of time spent in the in basket per physician per day. The numerator is the total number of minutes spent per physician in an in basket activity or navigator section within the reporting period. The denominator is the total number of days that the physicians logged in and worked during the reporting period.

^f^ Refers to count of unique patients among monthly patient encounters.

^g^ Refers to percentage of patients seen each month having an account in MyChart, the Epic patient health record platform.

For primary care, before COVID, PMARs were increasing by 0.023 messages per physician per day each month (95% CI, 0.015 to 0.031 messages; *P* < .001). In March 2020, PMARs increased by 1.940 messages per physician per day (95% CI, 1.508 to 2.371 messages; *P* < .001). During COVID, the numbers of PMARs did not change significantly (slope, −0.020; 95% CI, 1.508 to 2.370; *P* = .40). The rate of change in PMARs per clinical day was not significantly different during COVID compared with before COVID (slope, −0.040; 95% CI, −0.089 to 0.005; *P* = .08) (Figure).

For medical specialties, before COVID, PMARs were increasing by 0.033 messages per physician per day each month (95% CI, 0.026 to 0.039 message; *P* < .001). In March 2020, PMARs increased by 0.686 messages per physician per day (95% CI, 0.442 to 0.931 message; *P* < .001). During COVID, PMARs were increasing by 0.030 messages per physician per day each month (95% CI, 0.008 to 0.052 message; *P* = .009). The rate of change in PMAR per clinical day was not significantly different during COVID compared with before COVID (Figure).

For surgical specialties, before COVID, PMARs were increasing by 0.018 messages per physician per day each month (95% CI, 0.013 to 0.023 message; *P* < .001). In March 2020, PMARs did not change by a statistically significant amount. During COVID, PMARs were increasing by 0.072 messages per physician per day each month (95% CI, 0.043 to 0.101 message; *P* < .001). The rate of change in PMAR per clinical day was faster during COVID compared with before COVID (slope, 0.05; 95% CI, 0.024 to 0.083; *P* = .001) (Figure).

Consistent with national trends,^2^ during 2020 COVID months, monthly in-person visits decreased for all specialties (primary care, 17.1%; medical, 18.5%; surgical, 7.7%), whereas telehealth visits increased. Surgical specialties, however, experienced an increase in in-person visits between January and June 2021. The number of unique patients seeking care during the pandemic decreased for primary care and medical specialties through June 2021 but increased for surgical specialties in 2021.

## Discussion

Findings from this cross-sectional study indicate that during the first 15 months of the COVID-19 pandemic there was a small but sustained increase in patient message volume, in particular PMARs, across all specialties, despite a decrease (for primary care and medical physicians) in the number of patients seeking care during the same period.^3^ The increase in surgical patients in 2021 could be associated with clearance of backlog of elective surgeries delayed in 2020.^4^ The increase in electronic messages from patients did not displace patient calls. Limitations of this study include systematic underestimation of inbox time based on how Signal defines and captures inbox activity, which is limited to the time in the inbox screen and is not inclusive of time spent on other tasks necessary to address an inbox message (eg, phone calls or medical record review).^5^ However, burnout related to inbox burden is established.^1,6^ Given the existing physician burnout crisis and the already known pandemic-related stressors and risks to the physician workforce, the additional inbox burden reported here warrants additional exploration to assess the nature of pandemic-related medical advice requests and the generalizability of these findings. With COVID-19 potentially remaining a long-term endemic threat to public health, the priority to systematically address inbox burden before the pandemic through workflow redesign, team-based inbox management, and consideration of reimbursement for inbox-related work remains.

## Notes

Supplement. **eTable 1.** Epic Inbox Message Categories **eTable 2.** Physician Specialty Grouping
