# Supplementary material for: Trends in Electronic Health Record Inbox Messaging During the COVID-19 Pandemic in an Ambulatory Practice Network in New England
Source: JAMA Netw Open. 2021 Oct 12;4(10):e2131490. doi: 10.1001/jamanetworkopen.2021.31490 (PMC8511977; doi:10.1001/jamanetworkopen.2021.31490)
Supplement: Supplement. — eTable 1. Epic Inbox Message Categories eTable 2. Physician Specialty Grouping [file jamanetwopen-e2131490-s001.pdf]

Supplemental Online Content

Nath B, Williams B, Jeffery MM, et al. Trends in electronic health record inbox messaging during the COVID-19 pandemic in an ambulatory practice network in New England. *JAMA Netw Open*. 2021;4(10):e2131490. doi:10.1001/jamanetworkopen.2021.31490

eTable 1. Epic Inbox Message Categories
eTable 2. Physician Specialty Grouping

This supplemental material has been provided by the authors to give readers additional information about their work.

© 2021 Nath B et al. *JAMA Network Open.*

eTable 1. Epic Inbox Message Categories

Epic Inbox Message Categories	Message Categories by Source ofOrigin
Clinical Update Message	Patient Originated Messages
MyChart Patient Entered FlowsheetMessage	
Patient Call Back	
Patient Calls	
Patient CRM Request	
Patient History QuestionnaireSubmission	
Patient Medical Advice Request	
Patient Questionnaire Submission	
Patient Refill Request	
Patient Schedule Request	
Rx Auth	Prescription Renewal
Patient Reminder	Physician User Initiated
Results	
AC SIGNIFICANT WEIGHTCHANGE	System Generated
Anticoagulation Enrollment	
BestPractice	
Broadcast Message	
Canceled Orders	
Cardiology Message	
Case Message	
CE Event Notification	
Charts	
Clinical Letter	
Coding Query	
Completed Orders	
Covered Work	
E-Consult	
ED Charting Reminder	
Encounter Report	
E-Prescribing Errors	
Follow-up Reminder	
Help Desk	
Home Health Discharge	
Hospital ADT	
Hospital Chart Completion	
ID Chart Correction Message	

© 2021 Nath B et al. *JAMA Network Open.*

Incomplete Note	
Initiate Call Back	
INR Reminder	
IP Cosign Note	
IP ROUTING	
Letter Queue	
Media Manager	
Medication Cancellation	
Open Encounters	
Open Letters	
Overdue Message	
Overdue Rslt	
Overdue Rx Auth	
Post Mortem Notification	
Radiology Messages	
Refill Errors	
Rejected Transcription	
Reporting Workbench	
REVIEW REPORTS	
Route Plan	
Timeout Message	
Transcription	
Treatment Summary	
Unread Patient Message Notification	
Unsigned Orders	
Work Queue Message	
Addendum	Team Member Generated
CC Charts	
CE Outside Messages	
Chart Cosign	
Cosign - Clinic Orders	
Lab Add-ons	
Referral Letter Review	
Referral Message	
Referral Notification Letter	
Result Notes	
Rx Response	
Staff Message	

© 2021 Nath B et al. *JAMA Network Open.*

eTable 2. Physician Specialty Grouping

Physician Specialty	Combined Category
Family Medicine	Primary Care
Geriatrics	
Internal Medicine	
Neonatal-Perinatal Medicine (part of Pediatrics)	
Pediatrics	
Allergy	Medical Specialty
Cardiovascular Disease	
Child & Adolescent Psychiatry	
Dermatology	
Developmental-Behavioral Pediatrics	
Endocrinology, Diabetes & Metabolism	
Gastroenterology	
Infectious Diseases	
Nephrology	
Neurology	
Otolaryngology	
Pain Medicine	
Palliative Care	
Physical Medicine and Rehabilitation	
Physician Assistant	
Psychiatry	
Psychiatry & Neurology	
Pulmonary Critical Care Medicine	
Pulmonary Disease	
Rheumatology	
Sleep Medicine	
Colon & Rectal Surgery	Surgical Specialties
Foot & Ankle Surgery	
Gynecology	
Obstetrics and Gynecology	
Ophthalmology	
Orthopedic Surgery	
Podiatry	
Surgery	
Urology	

© 2021 Nath B et al. *JAMA Network Open.*
